# Conjugated Linoleic Acid and Its Beneficial Effects in Obesity, Cardiovascular Disease, and Cancer

**DOI:** 10.3390/nu12071913

**Published:** 2020-06-28

**Authors:** Sanjay Basak, Asim K. Duttaroy

**Affiliations:** 1 Molecular Biology Division, National Institute of Nutrition, Indian Council of Medical Research, Hyderabad 500007, India; sba_bioc@yahoo.com; 2 Department of Nutrition, Institute of Basic Medical Sciences, Faculty of Medicine, University of Oslo, 0317 Oslo, Norway

**Keywords:** conjugated linoleic acid, CLAcis-9,trans-11-(c9,t11)-CLA, *cis-12* (*t10c12*)-CLA, obesity, cancer, atherosclerosis

Conjugated linoleic acid (CLA) is a dietary polyunsaturated fatty acid found in animal fats such as red meat and dairy products [1]. Only trace amounts of CLA occur naturally in plant lipids, but various CLA isomers are produced during the chemical hydrogenation of fats [2]. While cis-9,trans-11-(c9,t11)-CLA is predominantly found in meat and dairy products, trans-9,trans-11-(t9,t11)-CLA is a constituent of vegetable oils. The isomers of CLA, *cis-9, trans-11* (*c9t11*)-CLA and *trans-10,* and *cis-12* (*t10c12*)-CLA are known to exert a variety of beneficial effects on the body [1,3]. CLA isomers are elongated and desaturated in a different pathway [4]. These isomers activate different nuclear receptors and, thus, they differentially regulate the expression of those genes related to lipid metabolism. Recently, den Hartigh [5] extensively reviewed the pre-clinical and human studies which have been conducted using CLAs. This work concluded that CLA has efficacy against cancer, obesity, and atherosclerosis. The involvement of gut bacteria was also highlighted. The Food and Drug Adminisrtation (FDA) in the USA defines *trans* fats as all unsaturated fatty acids that contain one or more nonconjugated double bond in a *trans* configuration. However, the CLA present in any food which contains conjugated *trans* fatty acids is not labelled as a *trans* fat. CLA is approved as being generally recognized as safe (GRAS) for a mixture of approximately 60–90% of the *cis*-9, *trans*-11, and *trans*-10,*cis*-12 isomers in about a 50:50 ratio. The normal CLA content of human plasma is approximately 0.1% of the total fatty acids [6]. The consumption of up to 6 g CLA/day for one year or 3.4 g CLA/day for up to 2 years is considered to be safe at this moment [7,8,9]. The mean daily intake of cis-9, trans-11 CLA is estimated to be 97.5 mg/d in the UK, which was calculated based on the daily intake of foodstuffs containing CLA isomers [10]. 

Conjugated linoleic acid (CLA) isomers offer a high stability in thermal processes, which ensures optimal meat quality after cooking [11]. Thus, it is expected that CLA will be added to a number of food products to improve health perspectives. However, there are few CLA-fortified foods available, indicating some of the unresolved issues of using CLA as a food ingredient [12]. Two primary challenges possibly limit the efficacies of CLAs, as den Hartigh [5] discussed in their review. Firstly, CLA isomers activate different nuclear receptors and the expression of the genes related to lipid metabolism. Since each isomer has specific effects, it is challenging to obtain isomer specific food products. Secondly, the majority of the studies have been conducted on the effects of the short-term supplementation of CLA on cardiovascular effects including hypertension [13]. Den Hartigh [5] has discussed these issues well in their review.

The anti-obesity effect of CLA has been the main focus of interest since it was reported in 1997 [14]. The overall results from human studies on the anti-obesity effects of CLA are somewhat weak compared with those from animal studies, as described by den Hartigh [5]. Several clinical trials have reported positive correlations between CLA supplementation and improvements in body mass index (BMI), body weight, body fat mass (BFM), abdominal adiposity, and lean body mass (LBM) [7,9,14,15,16,17]. Meta-analyses of three human studies concluded that CLA supplementation induced a significant reduction in body weight and BFM when 3.2–3.4 g/d CLA was supplemented for at least 6 months [7,8,17]. These results have encouraged the use of CLA in combination with other anti-obesity compounds and tools, such as an efficient delivery system that improves its efficacy. The anti-obesity effects of CLA are mediated via a reduced energy intake, increased energy expenditure, modulated metabolism in lipids, adipocytes and skeletal muscle [15]. The effects of CLA on food intake, which is independent of appetite-regulating neuropeptides in the hypothalamus, have not been conclusively proven [14,18]. However, CLA’s effect on food intake was suggested not to be responsible for the reduction of body fat in mice [19]. CLA increased the total energy expenditure in animal models but not in humans [20]. The effect of CLA on caloric intake in humans is controversial [16]. 

CLA has been suggested to prevent atherosclerosis in humans [21]. The anti-atherogenic effects observed with CLAs are presumably mediated not only by CLAs themselves but also by their metabolites [22]. A double-blind crossover human trial confirmed that consuming dairy products that are naturally enriched in cis-9, trans-11 (c9,t11) CLA by modification of cattle feed increases the concentration of this isomer in the plasma and cellular lipids of healthy men [23]. Conjugated linoleic acid improves blood pressure, a risk factor for cardiovascular disease (CVD), by increasing adiponectin and endothelial nitric oxide synthase activity [24]. Adipose tissue 9c,11t-CLA is associated with a lower risk of myocardial infarction. Meanwhile, 9c,11t-CLA, which is present in large amounts in the milk of pasture-grazed cows, might neutralize the adverse effects of the saturated fat content of dairy products. [25] t10c12-CLA activates 5’-adenosine monophosphate-activated protein kinase (AMPK) with concomitant increases in prostaglandin levels which are sufficient to cause lipid reductions in adipocytes [26]. The anti-steatotic effects of trans-10,cis-12 CLA, are potentially mediated via the increased lipid utilization by peripheral tissues [27]. CLA reduces hepatic steatosis and restores liver triacylglycerol secretion and the fatty acid profile during protein repletion in rats [28]

CLA has been shown to affect metabolism in both adipocytes and skeletal muscles [15,16]. CLA lowered the synthesis of lipids, adipogenesis and lipid storage in adipocytes but enhanced β-oxidation in muscles [29]. The *trans*-10,*cis*-12 isomer modulates the body’s composition, but the *cis*-9,*trans*-11 isomer has no effect [30]. Dietary *trans*-10,*cis*-12 CLA decreases the adiposity by stimulating the browning of adipocytes in mice [31,32]. However, further definitive studies are required to establish CLA as an anti-obesity agent for humans. Dietary CLA reduces plasma lipoproteins and early aortic atherosclerosis in hypercholesterolemic hamsters and is clinically demonstrated to be an anti-carcinogen in rodents. CLA has also been shown to regulate lipid metabolism and uptake by inhibiting lipoprotein lipase activity. Additionally, *9c11t-*CLA has been shown to stimulate the uptake of docosahexaenoic acid, 22:6n-3 (DHA), although the mechanism involved is not known [33]. 

*cis-12* (*t10c12*)-CLA can inhibit the growth of colon cancer cells and induce their death, whereas *c9t11*-CLA is known to mediate anti-carcinogenic effects via apoptosis. Many studies have been carried out on the roles of CLA in the prevention of cancers [12,15,17,34]. Studies on the effects of CLA on human cancers are lacking in any definitive conclusions [2,15,16]. Correlation data on the dietary intake of CLA or tissue CLA levels and the incidences of breast cancer incidences are inconsistent. So far, there is only one report of a CLA clinical trial for breast cancer [35]. CLA supplementation at least 10 days before the surgery was associated with reduced S14 levels in tumor tissue in patients with higher cancer scores (II), but not in patients with lower cancer scores (I) and no changes in the expression of fatty acid synthase or lipoprotein lipase. CLA decreased Ki-67 (tumor proliferation marker) without changing caspase 3 (an apoptosis marker) in these patients. This study concluded that CLA might be used in conjunction with the current options for breast cancer treatment. A negative correlation between the consumption of high-fat dairy and CLA and incidences of colorectal cancer was reported [36]. In another study, the supplementation of 3g CLA/day for 6 weeks in rectal cancer patients (Stage II-III) significantly reduced matrix metalloproteinase (MMP) 2 and MMP -9. CLA reduced angiogenesis and tumor invasion via the reduction of MMP-2 and MMP-9 [37,38]. CLA supplementation has been associated with reduced serum tumor necrosis factor-α (TNF-α), interleukin-1β (IL-1β), and C-reactive protein, suggesting that CLA may prevent inflammatory responses [37,38]. In fact, increasing evidence indicates that CLA inhibits new vessel growth in vitro and in vivo by reducing vascular endothelial growth factor (VEGF) and basic fibroblast growth factor, as well as by inhibiting the expression of the high-affinity VEGF receptor and fetal liver kinase-1 (flk-1) [39]. Fatty acids, especially n-3 fatty acids and CLAs inhibit, whereas n-6 fatty acid stimulated, angiogenesis in cancer cells [40]. However, cis-9,trans-11(c9,t11)-CLA was recently shown to stimulate angiogenesis in placental trophoblast cells [33].

A very small study suggested that CLA might prevent laryngeal papillomatosis, which is known to cause airway obstruction in young children [41]. All these human studies suggest the potential application of CLA in human cancers either alone or as a part of current anti-cancer treatments.

CLA has been shown to alleviate the adverse effects of immune stimulation, reducing inflammatory responses as well as hypersensitivity in animal models. Data suggest that CLA has anti-inflammatory effects and improves the innate immunity [42]. Dietary CLA reduced inflammation, and both c9t11-CLA and t10c12-CLA exhibited anti-inflammatory effects that reduced the inflammation associated with established collagen-induced arthritis in animal models [43]. Among CLA isomers, trans-10, cis-12 (t10c12)-CLA is known to participate in the modulation of pro-inflammatory cytokine secretion. t10c12-CLA probably modulates TNF-alpha production and NF-kappaB activation by a PPARgamma-dependent pathway in porcine peripheral blood mononuclear cells (PBMC) [44]. However, there are limited reports of CLA available with regard to improved immune responses in humans, which include suppressing allergic responses, enhancing antibody production following vaccination, reducing symptoms of atopic dermatitis, and rhinovirus infection [15,16,17,45,46,47]. Recent clinical studies have shown that CLA can cause a decrease in major pro-inflammatory markers, such as TNF-α or NFκB, even in rheumatoid arthritis patients [38,48]. The supplementation of CLA and vitamin E in combination significantly decreased markers for arthritis, such as citrullinated antibodies (CCP-A), MMP- 3, and white blood cell counts, in patients with rheumatoid arthritis [48]. CLA supplementation, along with the current treatment options, may have the potential to manage rheumatoid arthritis. The consumption of 4.5 g CLA/day for 12 weeks improved airway hyper-reactivity in overweight mild asthmatics, with a reduction in the leptin/adiponectin ratio [49]. CLA has also been shown to enhance the early stages of cutaneous wound healing by modulating oxidative stress and inflammatory responses [50]. CLA administration with 1% dietary calcium significantly improved the total ash percentages in femurs, confirming that CLA may be used to improve bone mass [51]. Dietary supplementation with CLA during suckling and extended to early infancy enhances the development of the intestinal mucosal immune (IgA) response in rats [52]. This enhancement of antibody synthesis in rats by feeding cis-9,trans-11 CLA during early life has also been demonstrated by others [52,53].

Current treatments for inflammatory bowel disease are limited. Therefore, there is a great need for better treatment options for this disease. There have been several investigations into the effects of CLA on inflammatory bowel disease [54,55]. CLA decreases adverse immune and inflammatory responses, suggesting CLA may be useful in reducing the symptoms of inflammatory bowel disease. The supplementation of 6 g CLA/day for 12 weeks decreased the Crohn’s disease activity index as well as improving inflammatory bowel disease and improving patients’ quality of life [54]. The intestinal gut microbiota is known to play a significant role in the development of inflammatory bowel disease as well [56]. CLA may modulate disease progression via modulating microbiota [56,57]. Since inflammatory bowel disease is associated with an increased risk of developing certain types of colorectal cancer, the application of CLA to control inflammatory bowel disease is of significant interest and might lead to a reduced risk of developing colorectal cancer.

The cis-9, trans-11 CLA isomer, which comprises about 40% of the commercial CLA mixture, acts as an active neuroprotective molecule. However, other isomers, such as trans-10, cis-12 CLA (40%), had no effects on neuroprotection. CLA significantly decreases angiogenesis in the cerebellum. The anti-angiogenic effect of CLA makes it a potential therapeutic adjuvant for the treatment of cancer and tumors in the brain [58]. CLA significantly increased neuronal Bcl-2 levels when added with glutamate and attenuated the glutamate-induced dissipation of the mitochondrial membrane potential, suggesting that it has a stabilizing influence on mitochondrial functions. [59] Isomer-specific effects of conjugated linoleic acid have also been reported on the proliferative activity of cultured neural progenitor cells [60].

Based on the current knowledge and evidence of CLA’s actions, CLA could be used to ameliorate several health issues. Therefore, not only the potential known adverse effects but also the unknown consequences should be closely monitored to ensure the proper application of CLA. CLA consists mainly of two isomers, *cis*-9,*trans*-11 and *trans*-10,*cis*-12, and their mixture has been approved for food as GRAS in the US since 2008. With ongoing applications in food production, CLA consumption is expected to rise, and the close monitoring of not only its efficacy but also its known and unknown consequences is required to ensure the proper application of CLA. The extensive review by den Hartigh [5] has provided us with an in-depth and up-to-date collection of information on CLAs. 
